# Impact of intensive care on renal function before graft harvest: results of a monocentric study

**DOI:** 10.1186/cc6120

**Published:** 2007-09-14

**Authors:** Valéry Blasco, Marc Leone, Julien Bouvenot, Alain Geissler, Jacques Albanèse, Claude Martin

**Affiliations:** 1 Département d'Anesthésie et de Réanimation, Hôpital Nord, Assistance Publique Hôpitaux de Marseille, Chemin des Bourrely, 13915 Marseille cedex 20, Université de la Méditerranée, Faculté de Médecine, 13005 Marseille, France; 2 Service de Biostatistique, Faculté de Médecine, Université de la Méditerranée, Bd Jean Moulin, 13005 Marseille, France

## Abstract

**Background:**

The aim of life-support measures in brain-dead donors is to preserve the functional value of their organs. In renal transplantation, serum creatinine level is one of the criteria for graft harvest. The aim of this study was to assess the impact of intensive care on donor renal function through two criteria: preharvesting serum creatinine level above 120 μmol/L and the elevation of serum creatinine level above 20% between intensive care unit (ICU) admission and graft harvest.

**Methods:**

Between 1 January 1999 and 31 December 2005, we performed an observational study on 143 brain-dead donors. ICU chronology, hemodynamic, hematosis, and treatment data were collected for each patient from ICU admission to kidney removal.

**Results:**

Twenty-two percent of the 143 patients had a serum creatinine level above 120 μmol/L before graft harvest. The independent factors revealed by multivariate analysis were the administration of epinephrine (odds ratio [OR]: 4.36, 95% confidence interval [CI]: 1.33 to 14.32; *p *= 0.015), oliguria (OR: 3.73, 95% CI: 1.22 to 11.36; *p *= 0.021), acidosis (OR: 3.26, 95% CI: 1.07 to 9.95; *p *= 0.038), the occurrence of disseminated intravascular coagulation (OR: 3.97, 95% CI: 1.05 to 15.02; *p *= 0.042), female gender (OR: 0.13, 95% CI: 0.03 to 0.50; *p *= 0.003), and the administration of desmopressin (OR: 0.12, 95% CI: 0.03 to 0.44; *p *= 0.002). The incidence of elevated serum creatinine level above 20% between admission and graft harvest was 41%. The independent risk factors were the duration of brain death greater than 24 hours (OR: 2.64, 95% CI: 1.25 to 5.59; *p *= 0.011) and the volume of mannitol (OR: 2.08, 95% CI: 1.03 to 4.21; *p *= 0.041).

**Conclusion:**

This study shows that the resuscitation of brain-dead donors impacts on their renal function. The uses of epinephrine and mannitol are associated with impairment of kidney function. It seems that graft harvest should be performed less than 24 hours after brain death diagnosis.

## Introduction

The success of organ transplantation depends on the quality of the resuscitation of donors [1]. However, its renal impact has not been subject to much evaluation up to the present. To the best of our knowledge, no studies have evaluated the impact of the resuscitation on the preharvesting renal function of potential brain-dead donors. The risk factors for renal function impairment in such patients are important since this can affect the future renal graft. Consequently, the primary objective of the present study was to assess the risk factors for renal impairment defined by a serum creatinine level above 120 μmol/L in a cohort of brain-dead donors. The secondary objective was to evaluate the risk factors for renal function deterioration, which was defined by a more than 20% rise of serum creatinine levels between intensive care unit (ICU) admission and graft harvest.

## Materials and methods

**Figure 1:**
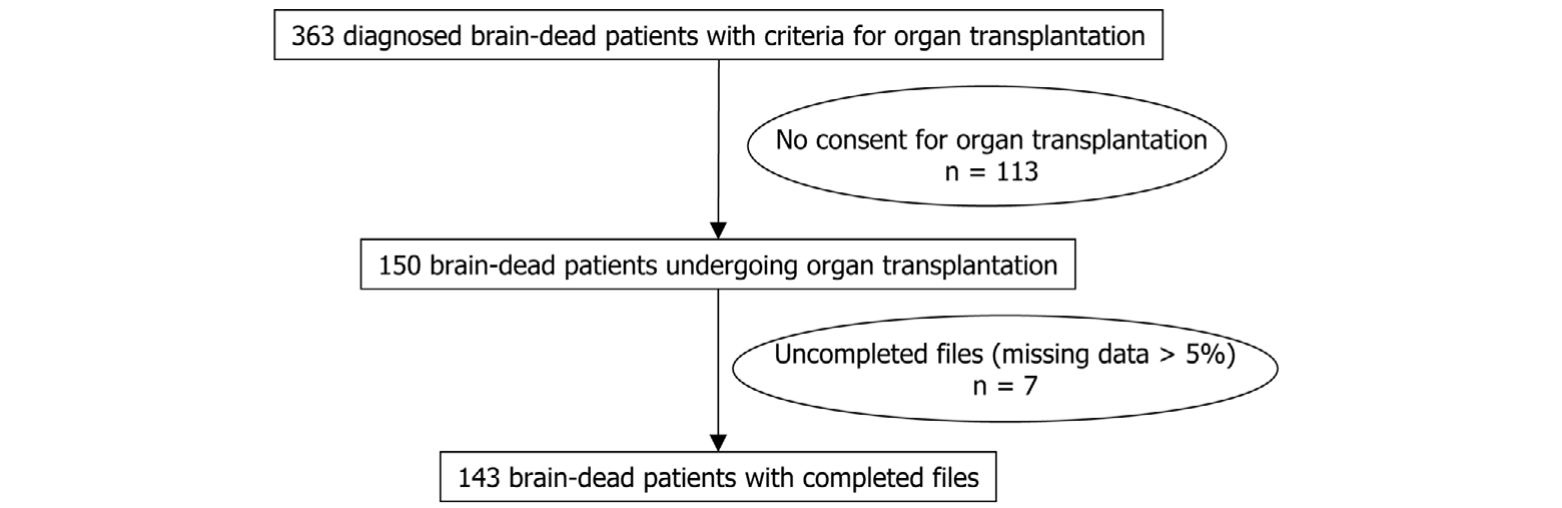
Flow chart of the inclusion.

Between 1 January 1999 and 31 December 2005, a retrospective observational study was conducted on 143 of 150 brain-dead donors admitted to a 16-bed medico-surgical ICU of an 800-bed university hospital (Hôpital Nord, Marseille, France) (Figure 1). Informed consent and approval by the ethics committee were waived due to observational nature of the study.

Computer data were collected prospectively by the physicians upon admission and during ICU stay. Physicians met weekly to complete the data after discharge. During data extraction, a software program performed a final check by eliminating aberrant values and suppressing duplications. The rate of uncompleted files was 5% (missing data: >5%). Patients with uncompleted files were excluded from the study. When the rate of missing data was less than 5%, they were ignored.

Donor resuscitation was performed according to standard clinical practices. Diagnosis of brain death was confirmed by the presence of a profound coma (flaccid, hypotonic, areactive) with no cerebral trunk reflex and the absence of ventilatory movement in a hypercapnic patient (PaCO_2 _[arterial partial pressure of carbon dioxide] of greater than 60 mm Hg) [2]. In accordance with French legislation, clinical diagnosis was confirmed by two electroencephalograms performed at least 4 hours apart or by angiography. As soon as the clinical diagnosis of brain death was confirmed, donor intensive care was performed according to French Society of Anesthesia and Intensive Care guidelines [3]. A written protocol, which is extracted from these guidelines, was distributed to all medical staff of our ICU.

Serum creatinine level is the most universal biological marker for estimating the glomerular filtration with a good prognostic value. Preharvesting serum creatinine level is considered to be an important determinant of renal function after transplantation [4]. Hence, the present study evaluated the impact of the resuscitation of brain-dead donors on renal function. The primary objective was to assess the risk factors associated with a preharvesting serum creatinine level above 120 μmol/L. To better characterize the impact of care provided in the ICU, the secondary objective was to identify the risk factors associated with a rise of more than 20% in serum creatinine levels between ICU admission and graft harvest. These two criteria have been reported in an article analyzing preoperative risk factors for acute postoperative renal failure [5,6]. The present study evaluated the influence of these two criteria on the renal graft quality through four criteria: delayed graft function, early acute rejection, return in dialysis (1 month, 1 year), and mortality (1 year). Delayed graft function was defined by the need for dialysis in the 7 days after transplantation [7]. Acute rejection of the renal allograft was defined by an elevation of serum creatinine levels of more than 20% between two successive measurements confirmed by a second biological screening and after elimination of another cause of graft dysfunction, which could be functional, toxic, urologic, or vascular. Any suspicion of acute rejection was confirmed by a histologic examination [8]. Data from donors were analyzed from ICU admission to kidney harvest. The demographic (gender and age) data, causes of ICU admission, duration of ICU stay, duration of shock, duration of brain death (from the clinical diagnosis), drugs used during the ICU stay (fluid expansion, catecholamines, osmotherapy, diuretics, and desmopressin), hemodynamic profile during ICU resuscitation, characteristics of renal function on admission and during ICU stay with special interest in oliguria (defined by a urine output of less than 0.5 mL/kg per hour for at least 2 consecutive hours), and creatinine serum levels were collected. Catecholamines have been used alone or in combination, as required, according to the attending physician.

Biological disseminated intravascular coagulation is defined by elevated D-dimers (D-dimers greater than 500 μg/L) and one major criterion for consumption of platelets or coagulation factors (platelet count of less than 50,000 mm^-3 ^or international normalized ratio of the prothrombin time of greater than 1.5) or two minor criteria for consumption of platelets or coagulation factors (platelet count of between 50,000 and 100,000 mm^-3 ^and international normalized ratio of the prothrombin time of between 1.2 and 1.5) [9]. Shock was defined by hypotension (systolic blood pressure of less than 90 mm Hg or a mean arterial pressure of less than 65 mm Hg) not reversed with fluid resuscitation and serum lactate level of above 3 mmol/L [10].

The collected data were entered into a Microsoft^® ^Office Excel 2000 spreadsheet (Microsoft Corporation, Redmond, WA, USA) and then were transferred to SPSS version 11.5.1.^® ^software (SPSS Inc., Chicago, IL, USA) for analysis of the results. Quantitative variables are presented in the form of mean ± standard deviation. Qualitative variables are expressed as percentages. For the univariate analysis, we determined the associations between serum creatinine level above 120 μmol/L or a rise of more than 20% in serum creatinine levels between ICU admission and organ harvest. The quantitative variables were assessed by a Student's *t *test or an analysis of variance. For the qualitative variables, a chi-square test or a Fisher exact test were used. For the multivariate analysis, the variables provided by univariate analysis were put into a logistic regression model. The values of successive models were evaluated by the Hosmer and Lemeshow test. The threshold for significance of the statistical tests was set at 5%.

## Results

Demographic characteristics and parameters of resuscitation are shown in Table 1. The age of patients was 38 ± 14 years. Males represented 62% of the study population. Head trauma (49%) and spontaneous intracranial bleeding (40%) accounted for the most frequent causes of death. Among these 143 donors, 31 (22%) had a serum creatinine concentration above 120 μmol/L. The significant risk factors associated with preharvesting serum creatinine level above 120 μmol/L are summarized in Table 1. The occurrence of disseminated intravascular coagulation and the occurrence of cardiac arrest, shock, or acidosis were statistically associated with a serum creatinine level above 120 μmol/L. For catecholamines, the use of epinephrine was associated with a serum creatinine level above 120 μmol/L. Substitutive opotherapy by desmopressin had no adverse effect on renal function. As shown in Table 2, six independent risk factors were retained by the logistic regression model (Hosmer-Lemeshow statistic: 0.96, with 85.3% of patients correctly identified by the model). The use of epinephrine during the donor resuscitation and the occurrence of oliguria, acidosis, and disseminated intravascular coagulation were significantly associated with a preharvesting serum creatinine level above 120 μmol/L. On the other hand, the administration of desmopressin and female gender were negatively correlated with a preharvesting serum creatinine level above 120 μmol/L. The rate of delayed graft function was significantly increased in the recipients from the donors with a serum creatinine level above 120 μmol/L as compared with those from donors with a serum creatinine level below 120 μmol/L. By contrast, there were no differences in the rates of acute rejection, return to dialysis, and mortality (Table 3).

**Table 1:** Factors for preharvesting serum creatinine level greater than 120 μmol/L

	All patients (*n *= 143)	Preharvesting creatinine	
		<120 μmol/L (*n *= 112)	>120 μmol/L (*n *= 31)
Demographic data			
Women, number (percentage)	54 (38)	50 (45)	4 (13)^a^
Age (years) (mean ± SD)	38 ± 14	38 ± 13	39 ± 15
Causes of ICU admission			
Head trauma, number (percentage)	70 (49)	50 (45)	20 (65)^a^
Intracranial bleeding, number (percentage)	57 (40)	53 (47)	4 (13)^a^
Cerebral anoxia, number (percentage)	8 (5.5)	4 (3.5)	4 (13)
Suicide, number (percentage)	8 (5.5)	5 (4.5)	3 (10)
ICU steps			
Duration of stay in ICU (hours) (mean ± SD)	70 ± 64	76 ± 66	49 ± 48^a^
Duration of brain death (hours) (mean ± SD)	30 ± 14	29 ± 12	33 ± 18
Duration of brain death >24 hours (percentage)	58 (41)	50 (44)	8 (26)
Catecholamines			
Dopamine, number (percentage)	37 (26)	32 (29)	5 (16)
Dobutamine, number (percentage)	11 (8)	10 (9)	1 (3)
Epinephrine, number (percentage)	51 (36)	31 (28)	20 (64)^a^
Norepinephrine, number (percentage)	101 (71)	80 (71)	21 (68)
Fluid expansion			
Isotonic saline solution (mL) (mean ± SD)	3,655 ± 4,003	3,667 ± 4,247	3,612 ± 3,015
Lactate ringer (mL) (mean ± SD)	2,582 ± 2,598	2,665 ± 2,633	2,280 ± 2,483
Gelatin (mL) (mean ± SD)	446 ± 928	466 ± 971	370 ± 763
Hydroxyethylstarch, number (percentage)	113 (79)	86 (77)	27 (87)
Hydroxyethylstarch (mL) (mean ± SD)	1,170 ± 1,080	1,140 ± 1,100	1,274 ± 1,015
Osmotherapy			
Mannitol 20% (mL) (mean ± SD)	185 ± 285	200 ± 301	132 ± 215
Hypertonic saline solution 7.5% (mL) (mean ± SD)	131 ± 296	130 ± 281	130 ± 351
Urine output modulators			
Furosemide, number (percentage)	36 (25)	30 (27)	6 (19)
Furosemide (mg) (mean ± SD)	16 ± 51	16 ± 56	12 ± 28
Desmopressin, number (percentage)	114 (80)	98 (87)	16 (52)^a^
Desmopressin (μg) (mean ± SD)	5.9 ± 5.7	6.8 ± 6.1	2.7 ± 3^a^
Hemodynamic profile during ICU resuscitation			
Cardiac arrest, number (percentage)	26 (18)	14 (12)	12 (39)^a^
Shock, number (percentage)	93 (65)	67 (60)	26 (83)^a^
Duration of shock (minutes) (mean ± SD)	80 ± 142	61 ± 104	150 ± 223^a^
MAP upon admission (mm Hg) (mean ± SD)	89 ± 25	93 ± 23	75 ± 26^a^
Preharvesting MAP (mm Hg) (mean ± SD)	81 ± 17	82 ± 18	77 ± 16
Respiratory profile during ICU resuscitation			
Acute respiratory distress syndrome, number (percentage)	53 (37)	37 (33)	16 (52)
Acute lung injury, number (percentage)	33 (23)	30 (27)	3 (10)^a^
Characteristics of renal function			
Oliguria, number (percentage)	66 (46)	44 (39)	22 (71)^a^
Serum creatinine upon admission (μmol/L) (mean ± SD)	89 ± 38	79 ± 26	125 ± 49^a^
Preharvesting serum creatinine (μmol/L) (mean ± SD)	98 ± 61	75 ± 21	180 ± 83^a^
Acidosis (pH <7.30), number (percentage)	45 (31)	24 (21)	21 (68)^a^
Disseminated intravascular coagulation, number (percentage)	24 (17)	13 (12)	11 (35)^a^
Injection of contrast, number (percentage)	97 (68)	77 (69)	20 (64)

^a^*p *< 0.05. ICU, intensive care unit; MAP, mean arterial pressure; SD, standard deviation.

**Table 2:** Independent risk factors for preharvesting serum creatinine level greater than 120 μmol/L

	*P *value	Odds ratio	95% confidence interval
Epinephrine use	0.015	4.36	1.33–14.32
Disseminated intravascular coagulation	0.042	3.97	1.05–15.02
Oliguria	0.021	3.73	1.22–11.35
Acidosis	0.038	3.26	1.07–9.95
Female gender	0.003	0.13	0.03–0.50
Desmopressin use	0.002	0.12	0.03–0.44

**Table 3:** Kidney complications after transplantation

Complications	All patients (*n *= 233)	Preharvesting serum creatinine >120 μmol/L (*n *= 51)	Elevated serum creatinine >20% (*n *= 94)
Delayed graft function, number (percentage)	88 (38)	29 (57)^a^	35 (37)
Acute rejection, number (percentage)	36 (15.5)	9 (8)	19 (20)
Return in dialysis at 1 month, number (percentage)	8 (3.4)	2 (4)	2 (2.1)
Return in dialysis at 1 year, number (percentage)	14 (6)	3 (6)	4 (4.3)
Mortality at 1 year, number (percentage)	6 (2.6)	4 (7.8)	2 (2.1)

^a^*p *< 0.05.

A rise of more than 20% in serum creatinine levels between ICU admission and graft harvest was observed in 58 (41%) patients (Table 4). This rise was detected in the patients who were treated with a large volume of mannitol (276 ± 241 mL versus 123 ± 221 mL; *p *= 0.003), in whom the duration of brain death was above 24 hours (76% versus 53%; *p *= 0.006) and in whom an iodinated radiographic contrast was injected (78% versus 61%; *p *= 0.04). When multivariate logistic regression analysis was applied (Hosmer-Lemeshow statistic: 0.95, with 64.1% of the patients correctly identified by the model), the volume of mannitol infused during the initial resuscitation (odds ratio [OR]: 2.08, 95% confidence interval [CI]: 1.03 to 4.21; *p *= 0.04) and duration of brain death greater than 24 hours (OR: 2.64, 95% CI: 1.25 to 5.59; *p *= 0.01) were associated with a rise of more than 20% in serum creatinine concentrations. The rise of more than 20% in serum creatinine levels was not associated with significant changes in the rates of delayed graft function, acute rejection, return to dialysis, and mortality (Table 3).

**Table 4:** Factors for an elevation of serum creatinine levels of 20% or more

	Elevation of serum creatinine levels	
	<20% (*n *= 85)	>20% (*n *= 58)
Demographic data		
Women, number (percentage)	36 (42)	18 (31)
Age (years) (mean ± SD)	39 ± 14	38 ± 13
Causes of ICU admission		
Head trauma, number (percentage)	37 (43)	33 (57)
Intracranial bleeding, number (percentage)	37 (44)	20 (36)
Cerebral anoxia, number (percentage)	6 (8)	2 (3)
Suicide, number (percentage)	5 (6)	3 (5)
ICU steps		
Duration of stay in ICU (hours) (mean ± SD)	63 ± 56	80 ± 72
Duration of brain death (hours) (mean ± SD)	27 ± 11	34 ± 17^a^
Duration of brain death >24 hours, number (percentage)	45 (53)	44 (76)^a^
Catecholamines		
Dopamine, number (percentage)	26 (31)	11 (19)
Dobutamine, number (percentage)	5 (6)	6 (10)
Epinephrine, number (percentage)	28 (33)	23 (40)
Norepinephrine, number (percentage)	58 (68)	43 (74)
Fluid expansion		
Isotonic saline solution (mL) (mean ± SD)	3,406 ± 3,825	4,020 ± 4,259
Lactate ringer (mL) (mean ± SD)	2,696 ± 2,641	2,413 ± 2,546
Gelatin (mL) (mean ± SD)	441 ± 930	452 ± 933
Hydroxyethylstarch, number (percentage)	67 (79)	46 (79)
Hydroxyethylstarch (mL) (mean ± SD)	1,047 ± 1,019	1,349 ± 1,148
Osmotherapy		
Mannitol 20% (mL) (mean ± SD)	123 ± 221	276 ± 241^a^
Hypertonic saline solution 7.5% (mL) (mean ± SD)	111 ± 235	159 ± 369
Urine output modulators		
Furosemide, number (percentage)	19 (22)	17 (29)
Furosemide (mg) (mean ± SD)	14 ± 57	17 ± 42
Desmopressin, number (percentage)	68 (80)	46 (79)
Desmopressin (μg) (mean ± SD)	5.7 ± 6.2	6.2 ± 5.1
Hemodynamic profile		
Cardiac arrest, number (percentage)	16 (19)	10 (17)
Shock, number (percentage)	53 (62)	40 (69)
Duration of shock (minutes) (mean ± SD)	65 ± 108	102 ± 180
MAP upon admission (mm Hg) (mean ± SD)	91 ± 25	87 ± 25
Preharvesting MAP (mm Hg) (mean ± SD)	81 ± 17	81 ± 18
Respiratory profile		
Acute respiratory distress syndrome, number (percentage)	22 (26)	23 (40)
Acute lung injury, number (percentage)	21 (25)	11 (19)
Characteristics of renal function		
Oliguria, number (percentage)	39 (46)	27 (47)
Serum creatinine upon admission (μmol/L) (mean ± SD)	95 ± 42	80 ± 28^a^
Preharvesting serum creatinine (μmol/L) (mean ± SD)	82 ± 40	121 ± 76^a^
Acidosis (pH <7.30), number (percentage)	23 (27)	22 (38)
Disseminated intravascular coagulation, number (percentage)	11 (13)	13 (22)
Injection of contrast, number (percentage)	52 (61)	45 (78)^a^

^a^*p *< 0.05. ICU, intensive care unit; MAP, mean arterial pressure; SD, standard deviation.

## Discussion

To the best of our knowledge, no studies have compared the impact of resuscitation on renal function before graft harvest. Brain death is associated with complex hemodynamic, endocrine, and metabolic dysfunction that can lead to major complications with the potential donor. Untreated, this can progress to cardiovascular collapse with loss of valuable organs for transplantation. However, drugs used have an adverse potential effect on preharvesting renal function.

The present study confirms that elevated preharvesting serum creatinine levels are associated with an increased rate of delayed graft function [11]. Hence, we sought to determine the factors associated with serum creatinine levels above 120 μmol/L in the donors. The administration of epinephrine is an independent risk factor associated with a rise in serum creatinine level above 120 μmol/L. This risk factor has not been previously described. The use of epinephrine induces a renal vasoconstriction [12]. This can also reflect a profound state of hemodynamic instability. In agreement with our result, a recent study showed that the use of epinephrine in donors was associated with a negative influence on the graft quality after transplantation [13].

The occurrence of disseminated intravascular coagulation is an independent risk factor associated with a serum creatinine level above 120 μmol/L. The link between hemostasis and brain injury has been reported elsewhere [14]. In cases of cerebral injury, one can observe coagulation disorders resulting in disseminated intravascular coagulation [15]. Also, the occurrence of acidosis is an independent risk factor, probably reflecting a cellular dysoxia.

The occurrence of oliguria is an independent risk factor associated with a serum creatinine concentration above 120 μmol/L. Oliguria can be a marker of hemodynamic instability or acute renal failure. This risk factor has been described in recipients but not in donors [16]. Oliguria, whatever its significance, should be avoided in potential donors. However, in our study, the volume of fluid resuscitation did not impact on the value of preharvesting serum creatinine level. This suggests that an aggressive volume resuscitation in order to avoid oliguria is not always associated with clinical success.

Administration of desmopressin was inversely correlated with the occurrence of a serum creatinine level above 120 μmol/L. The effects of desmopressin on graft function are variable, and several studies have reported no changes in renal function [17]. By contrast, the impact on pancreas grafts is deleterious, with microthromboses and loss of function [18]. One possible protective mechanism at the renal level may be a vasodilatation obtained via the activation of V2 receptors. Indeed, desmopressin induces a vasodilatation via the production of nitric oxide [19].

Although the admission serum creatinine levels are significantly higher in the group with a preharvesting serum creatinine level above 120 μmol/L, this factor is not found as an independent risk factor. By contrast, the lower preharvesting serum creatinine level in females can be the consequence of their lower muscle mass. The analysis of estimated glomerular filtration rate instead of serum creatinine levels would resolve this ambiguity.

A rise of more than 20% in serum creatinine levels between ICU admission and graft harvest, with an incidence of 41%, is associated with a duration of brain death of greater than 24 hours. A prior study found that the duration of resuscitation does not influence the quality of kidney grafts transplanted if the hemodynamic condition of the donor is maintained [20]. However, the link between the quality of kidney graft and the ICU length of stay appears to be complex. Prolonged ICU stay of the donor has been shown to be correlated with a lower risk of delayed graft function in the recipients [13]. In regard to our results, a long duration of ICU stay before the occurrence of brain death does not affect the quality of kidney, whereas a prolonged duration of brain death may impair the preharvesting renal function. Hence, the duration of brain death should be shortened as much as possible in order to preserve the renal function.

This rise is also associated with the use of a large volume of mannitol. Mannitol increases urine output but does not reduce the incidence of acute renal failure [21]. Cases of acute renal failure can be encountered in relation to mannitol serum levels that are too high [22,23]. One hypothesis is that mannitol infusion could generate osmotic nephrosis-like lesions with a direct nephrotoxic effect [24]. Interestingly, the use of hypertonic saline solution, which is an alternative to mannitol [25], is not associated with a worsening of renal function in our patients.

We acknowledge that the present study has several limitations. The retrospective design limits the interpretation of data. In addition, the patients were hospitalized in a single institution, which reflects a local policy of management of donors. Lastly, to define the worsening of renal function, we used a criterion that is not precisely described in the literature in the field of renal transplantation. In fact, the definition of acute renal failure is far from consensus [26]. One can note that our criteria for evaluating renal function are restrictive.

## Conclusion

In summary, within the limitations of this study, the use of epinephrine in the potential donors is associated with an increased risk (by a factor of 4.3) of preharvesting serum creatinine level above 120 μmol/L. A large volume of mannitol is associated with an increased risk (by a factor of 2) of a rise of more than 20% in serum creatinine levels between ICU admission and graft harvest, whereas the use of hypertonic saline solutions does not share this negative effect. Importantly, although the duration of ICU stay prior to brain death occurrence has no impact on the preharvesting renal function, the transplantation procedure should be performed as soon as the brain death is detected. Lastly, administration of desmopressin is associated with a preservation of renal function. This result deserves to be investigated in further prospective studies.

## Key messages

• The present study was aimed at assessing the impact of intensive care on donor renal function.

• The use of epinephrine in the potential donors is associated with an increased risk (by a factor of 4.3) of a preharvesting serum creatinine level above 120 μmol/L.

• A large volume of mannitol is associated with a twofold risk of a rise of more than 20% in serum creatinine levels between ICU admission and graft harvest, whereas the use of hypertonic saline solutions does not share this effect.

• Although the duration of ICU stay prior to brain death occurrence has no impact on the preharvesting renal function, the transplantation procedure should be performed as soon as the brain death is detected. Administration of desmopressin is associated with a preservation of renal function.

## Abbreviations

CI = confidence interval; ICU = intensive care unit; OR = odds ratio.

## Competing interests

The authors declare that they have no competing interests.

## Authors' contributions

JA and CM conceived and supervised the study, interpreted results, and drafted the manuscript. VB and ML conducted searches, abstracted data, corresponded with authors, analyzed and interpreted results, and edited the manuscript. AG provided data on the recipient kidney function. JB advised on statistical analyses, interpreted results, and drafted the manuscript. All authors read and approved the final manuscript.
